# An appraisal of natural products active against parasitic nematodes of animals

**DOI:** 10.1186/s13071-019-3537-1

**Published:** 2019-06-17

**Authors:** Jose F. Garcia-Bustos, Brad E. Sleebs, Robin B. Gasser

**Affiliations:** 1 Faculty of Veterinary and Agricultural Sciences, The University of Melbourne, Parkville, Victoria 3010 Australia; 2 Walter and Eliza Hall Institute of Medical Research, Parkville, Victoria 3052 Australia; 3 Faculty of Medicine, Dentistry and Health Sciences, The University of Melbourne, Parkville, Victoria 3010 Australia

**Keywords:** Animals, Anthelmintics, Natural products, Nematocides

## Abstract

Here, the scientific and patent literature on the activities of purified natural compounds has been reviewed, with the aim of assessing their suitability as anthelmintic drug discovery starting points. Only compounds described as active against parasitic nematodes of animals or against the model nematode *Caenorhabditis elegans* have been analysed. Scientific articles published since 2010 and patents granted from 2000, both inclusive, have been included in this analysis. The results show a scarcity of novel chemical structures, a limited follow-up of compounds disclosed before 2010 and a bias towards the screening of plant products, almost to the exclusion of other sources, when microbial extracts have, historically, provided most starting points for anti-infective drugs. All plant products published in this period were previously known, alerting to the high re-discovery rates of a limited number of chemical classes from this source. The most promising compounds described in the literature reviewed here, namely the linear nemadectin-derivatives, are novel and of bacterial origin. Patented but otherwise unpublished spiroketal structures also appear as interesting scaffolds for future development. The patent literature confirmed that it is possible to patent derivatives of previously known products, making them valid starting points for translational research.

## Background

Parasitic helminths of humans and other animals cause diseases of major socio-economic importance globally. In particular, parasitic nematodes have a massive, long-term impact on human health and cause substantial suffering, particularly in children [1]. The World Health Organization (WHO) estimates that ~ 1.5 billion people were infected with intestinal worms in 2018, predominantly in disadvantaged communities [2]. The disease burden caused by these parasitic worms is similar to liver cancer and higher than prostate cancer [3]. Parasitic worms also cause substantial morbidity and mortality in domesticated and wild animals worldwide, and major losses to the global food production annually [1]. For instance, nematodes of the order Strongylida cause some of the most important diseases of livestock worldwide, affecting hundreds of millions of food animals (including sheep, goats, cattle and pigs), with economic losses estimated at tens of billions of dollars per annum globally (cf. [4]).

The livestock industry plays a major role in the economies of both developed and developing countries. The production of livestock animals provides food, animal products (e.g. leather, hides and wool), income, employment, a source of organic fertilizer and biogas as well as draught and work power (e.g. in countries where modern machinery is unaffordable or unavailable). In the developing world, the greatest impact of parasitic infections and diseases relates to productivity losses and lost socioeconomic potential. In the developed world, on the other hand, the main impact relates to the cost of parasite control [5, 6] associated with animal management and pasture utilisation, the use of anthelmintics and strategic or integrated anthelmintic treatment programs (cf. [7]).

**Fig. 1:**
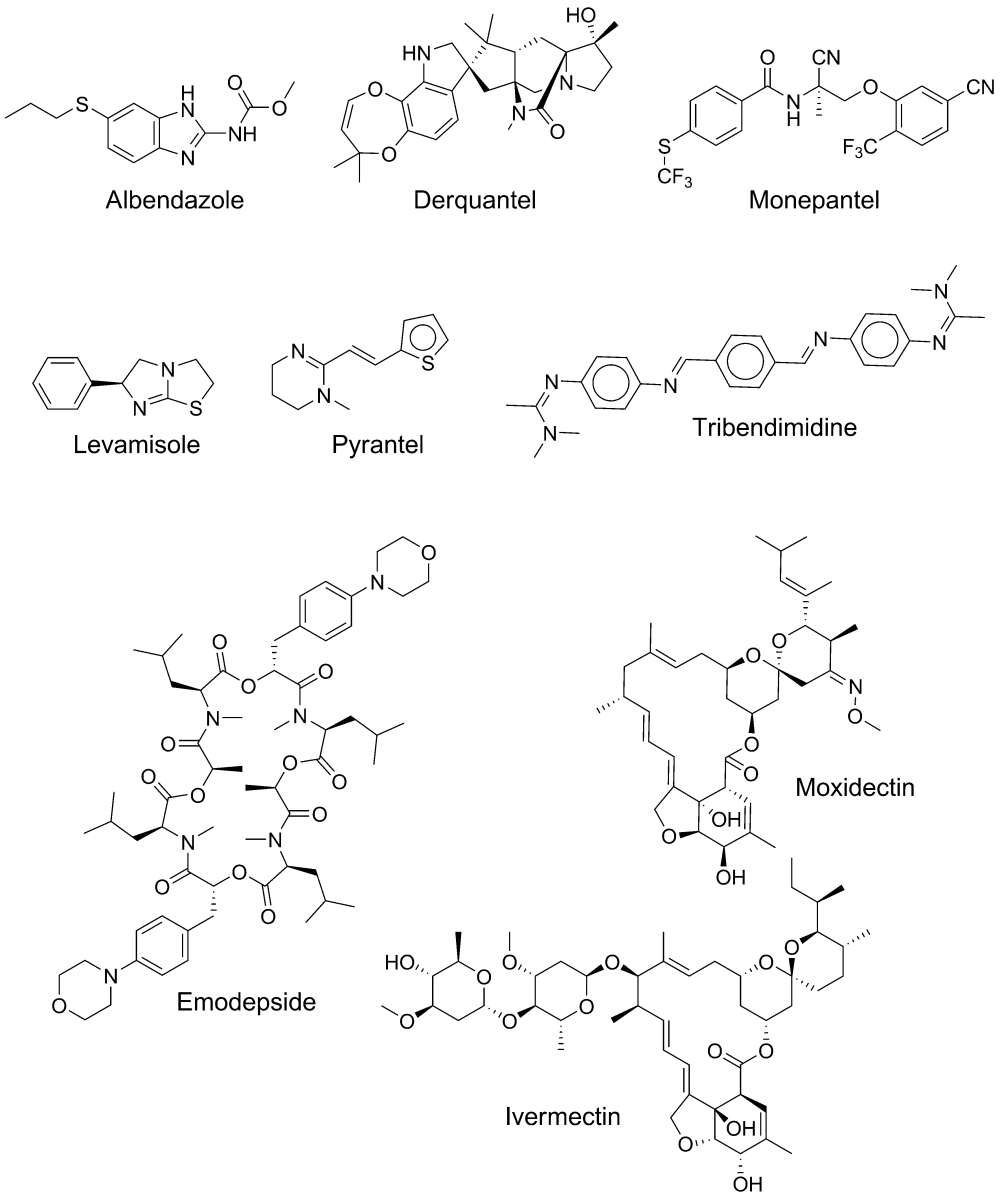
Examples of currently used nematocides

Different species of parasitic nematodes vary markedly in their pathogenicity, geographical distribution and susceptibility to anthelmintic drugs [8]. Mixed infections, involving multiple genera and species are common, and often have a greater impact on the host than monospecific infections. Moreover, the species composition of the parasites infecting an animal can have an important bearing on the severity of infection or disease [9], and the varied pathological effects on their host contribute to a decreased production performance. Control of animal-parasitic nematodes relies heavily on the treatment with anthelmintics (Fig. 1). Such drugs include aminoacetonitrile derivatives (e.g. monepantel), aminophenylamidines (tribendimidine), benzimidazoles (albendazole), imidazothiazoles (levamisole), macrocyclic lactones (ivermectin and moxidectin), spiroindoles (derquantel) and tetrahydropyrimidines (morantel, oxantel and pyrantel) [10, 11].

Pyrantel and levamisole act by binding to nematode-specific acetylcholine receptor ion channels in nerves and muscles of nematodes, resulting in a paralysis or spastic muscle contraction in worms [12]; thus, the worms are unable to move normally and are removed from the gastrointestinal tract by peristalsis. Benzimidazoles work against a range of nematode species [13] by binding to tubulin (cytoskeletal protein) [14] and thus block the formation of the microtubules essential for critical processes in cell physiology, such as intracytoplasmic transport and cell division. Macrocyclic lactones act by opening glutamate-gated chloride channels, increasing the flow of chloride ions and leading to defects in neurotransmission and flaccid paralysis (cf. [12]). Novel classes of anthelmintics, including an aminoacetonitrile (monepantel), a cyclooctadepsipeptide (emodepside) and a spiroindole (derquantel), have become available relatively recently [10, 11]. Their mode of action involves binding to neural acetylcholine receptors (monepantel) [15], to voltage-/calcium-dependent potassium channels and to G-protein-coupled latrophilin-like receptors (emodepside) [16] or B-subtype nicotinic acetylcholine receptors in muscle cells (derquantel) [17], resulting in spastic (monepantel) or flaccid (emodepside and derquantel) paralysis of sensitive nematodes, leading to death or elimination from the host animal.

The ease of administration (enteral or parenteral) and relatively low costs of current anthelmintic drugs has led to their extensive use in animals and, consequently, to the emergence of resistances. Indeed, resistances in nematodes of livestock animals to benzimidazoles, imidazothiazoles/tetrahydropyrimidines and macrocyclic lactones are widespread and have been reported to occur on all continents (reviewed in [18–20]).

Given the impact of nematode infections on animal and human populations and the spread of anthelmintic resistance, there is an urgent need to discover and develop new drugs for the sustained and effective control of nematodes into the future. Although monepantel has provided some impetus for the design/development of new classes of nematocides and anthelmintics in general, resistance to this compound appeared quickly and is now emerging [21]. Compounding the problem, success in discovering novel anthelmintics using diverse screening approaches has been limited in the last decade. In Australia, monepantel was registered under the trade name Zolvix for treatment of sheep in 2010 and, since then, only combinations of two or three previously used active components have been marketed. The exception is a combination of abamectin with a truly novel anthelmintic, derquantel (a semi-synthetic paraherquamide) [22], which was registered under the name Startect in 2014.

In the present work, we have critically reviewed published articles (from 2010) and patents (from 2000) describing the activity of pure natural products against nematodes able to parasitise animals and humans, focusing on their suitability as chemical starting points for anthelmintic drug discovery efforts. We have also included compounds tested only against the free-living nematode *Caenorhabditis elegans*, because this organism is often used as a model in which to study the mode of action of compounds active against parasitic nematodes. Reports that dealt exclusively with other free-living or plant-parasitic nematodes have not been included.

## Challenges and opportunities in the development of natural products as drugs

Derquantel is one of the latest compounds in a long tradition of natural products at the origin of most anti-infective drugs, with the exception of anti-virals and, to some extent, anti-parasitics. Nevertheless, the pharmacognosy of helminth infections is relatively rich. Traditional medicine in most countries includes natural remedies against parasitic worms, and the comprehensive review by Ghisalberti [23] lists anthelmintic compounds extracted from different, but mostly plant sources. However, directly chewing plant parts or drinking infusions thereof represent highly irreproducible treatments. Plants produce different metabolites that are present at different concentrations, depending on the planting season, environmental temperature and/or rainfall levels during growth periods, vitality of different plant parts and degree of predation by herbivores, all of which are variable and demand optimisation if reproducible product yields are to be obtained. The cost and effort of such an optimisation might be achievable and sustainable for high value crops, such as *Artemisia annua*, the source of the first-line antimalarial compound, artemisinin [24, 25]. For less well-funded or lower demand areas, identifying and extracting pure active compounds is the only approach to achieving a controlled and consistently efficacious product. The process may, however, disrupt useful interactions among components in crude extracts, some of which may enhance curative potency or bioavailability. That is generally an unavoidable loss, because such synergistic effects are usually inconsistent due to the variable ratios of interacting compounds present in the source, and the chance of synergy has to be balanced against the benefits of purification that eliminates undesirable and toxic compounds frequently found in natural extracts and which are well-known and documented for widely used herbal remedies (cf. [26, 27]). Contrary to popular belief, not all natural compounds are innocuous and much less beneficial, as many potently toxic compounds are made naturally by living organisms (cf. [28]).

The above notwithstanding, natural products also have distinct advantages. Arguably, their structures have evolved during millions of years to produce a defined biological effect on a living organism, be it a competitor, a grazer or a prey organism. When this effect happens to be useful to humans, we have a ready-made chemical structure with the capacity to cross biological barriers and survive or even co-opt mammalian metabolism to reach and modulate the activity of its pharmacological target. Since evolution hardly ever follows a linear path, the selected chemical structures tend to be complex and tri-dimensional, very different from those readily accessible through synthetic chemistry and hence contributing much needed structural diversity and novel chemical starting points to drug design [29].

However, to make a natural compound available in a pure form for pharmaceutical development is nontrivial. The compound needs to be isolated first and its chemical structure has to be elucidated, at the very least for quality control purposes and frequently also for synthetic reasons, when it cannot be economically sourced from its producer. Identifying the active components in a natural extract is usually laborious, and success does not guarantee product availability. The original compound is hardly ever optimal in terms of toxicity or pharmacokinetic properties, and it usually has a complex chemical structure. The latter property hinders chemical optimisation and can make total synthesis with useful yields impossible in practice with existing technology. That means that the starting material has to be obtained directly from natural sources. When the source is a microbe that can be cultured on a large scale, the product can usually be made reproducibly and cheaply enough that it, or slightly modified semi-synthetic versions, can succeed in clinical applications, as has been the case for most antibacterials [29]. In contrast, compounds found exclusively in plants present a range of cost and sustainability issues, depending, for example, on whether the producing species can be cultivated to yield one or more harvests per year, or rather the compound is found in a slow-growing plant, perhaps even in tissues that cannot be sustainably harvested, such as root bark. An even less favourable case is that of natural products found in rare marine metazoan organisms. When the compound’s application is forecasted to generate high economic returns, as is the case for the anti-cancer compound ET-743, complex and costly processes are developed to farm the producing organism (a sea squirt, *Ecteinascidia turbinata*) in large aquaria or marine farms [30]. Anthelmintics do not generate high economic returns, and low-cost total synthesis is usually the only path forward to commercial development. The ET-743 scenario, however, provides a small glimmer of hope for the sustainable sourcing of low-cost natural products from metazoans. In this, as well as in other studied cases, the biosynthetic capacity to make these complex structures is usually not part of an animal’s metabolic repertoire. The active compounds are produced by microbial endosymbionts that can, in principle, be isolated and grown in the laboratory [31], keeping in mind, however, that the fastidious nature of most endosymbiotic microorganism can make this more a theoretical possibility than a practical scenario.

Natural products, like any other drug lead, usually need to be chemically modified to improve properties such as aqueous solubility, activity spectrum, pharmacokinetic properties or chemical stability. This job falls on medicinal chemists, whose typical reaction on seeing a natural structure is to make it abundantly clear that it is not “drug-like”. Drug likeness, however, is still a collection of physicochemical attributes with numerous exceptions and parameter choices heavily influenced by a chemist’s professional experience [32]. The concept is generally attributed to Christopher Lipinski, who analysed the physicochemical properties common to most synthetic drugs orally efficacious in humans and summarised them as a “rule-of-five” [33]. Lipinski and other workers [34, 35] later refined the drug-likeness rules to include more molecular descriptors, but most authors explicitly admit that drug-likeness criteria are generalisations that do not apply to all drug development scenarios, especially to natural products, which seem to follow their own rules and generally occupy a different section of chemical space relative to “drug-like” synthetic compounds. Hence, drugs, particularly anti-infective compounds other than anti-virals, are discovered much more frequently than they are designed, with anti-infective discovery being additionally hampered by the scarcity of natural product-like compounds in corporate screening libraries. Interestingly, many natural compound libraries have been spun-off by pharmaceutical companies and have found a home at biotechnology companies and academic centres, where they may be able to power future drug discovery outside of large corporations [36, 37].

The concept of “drug-likeness” has an additional twist in the case of anthelmintics. It should be remembered that the rules sought to encapsulate properties common to orally absorbed drugs, but compounds required to eliminate gastrointestinal worms might not need to be absorbed into the blood circulation, at least not in principle. This would lift a large part of the drug safety burden, as poorly absorbed compounds are much less likely to cause systemic toxicities. However, many parasitic nematodes infect tissues outside of the gastrointestinal tract, and the requirement for a broad spectrum of action can negate the above advantage. Currently, it is also unclear whether a product aimed at treating gastrointestinal blood-feeding nematodes, such as hookworms or *Haemonchus contortus*, would need to be present at therapeutic levels in blood in order to be fully efficacious.

In summary, the unique structures of pharmacologically active natural products endow them with many of the desirable properties of a drug, but they also make them difficult to modify chemically. Natural products tend to be of comparatively larger molecular size and higher structural complexity than most synthetic drugs in use, with multiple functional groups that need to be protected and de-protected during semi-synthesis, and often incorporate chiral centres that generate multiple enantiomers upon modification, only one of which is the desired product. Chirality often introduces the need for costly purification procedures to avoid dosing subjects with a mixture of enantiomers, in which all but one compound will be useless at best, or toxic at worst. It is these chemical properties that have reduced the contribution of natural products to drug lead optimisation programmes and have led most pharmaceutical companies to close down or spin-off their natural product R&D groups, explaining in part the current dearth of novel anti-infective leads.

## Chemical classes of natural compounds described as anthelmintics

### Phenols

**Fig. 2:**
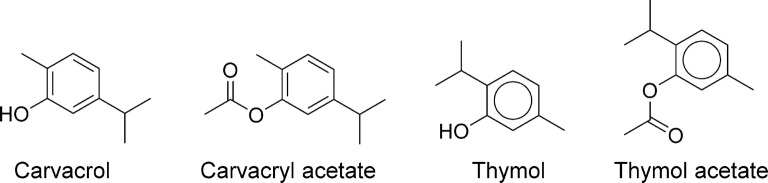
Phenols

**Fig. 3:**
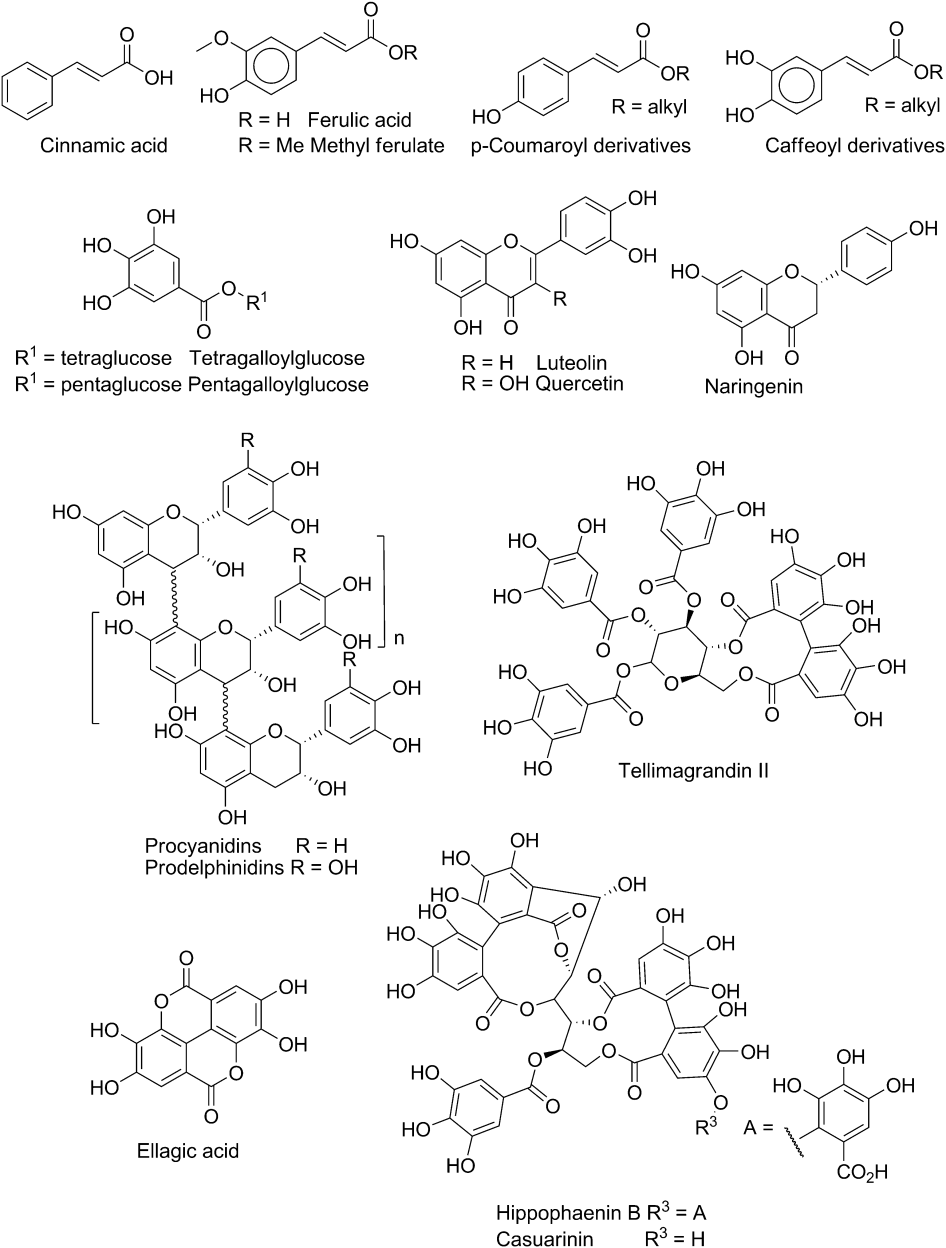
Cinnamoyl derivatives and polyphenols (tannins, flavonoids and isoflavonoids)

Phenolic compounds are widespread in nature and are often components of natural product extracts. They include monophenols or terpenoids (thymols, cresols and carvacrol) (Fig. 2), benzene diols (catechols and resorcinols), flavonoids (e.g. quercetin) (Fig. 3), substituted benzoic acid (gallic acids and vanillic acids) and cinnamic acids. Many of these classes frequently appear in the natural product literature, particularly in studies using extracts of plants and in organisms hosting symbionts able to photosynthesize. Photosynthesis underpins key synthetic routes, such as the acetate-malonate and shikimate pathways that generate the malonyl-CoA and shikimic acid synthons utilised in the biosynthesis of a large number of phenolic compounds [38].

The reported biological activities of natural product extracts commonly result from the intrinsic antioxidant character of phenolic compounds. This property originates from the ability of the phenol group to form a phenoxy radical upon donating a hydrogen atom. This phenoxy anion can scavenge free radicals and participate in metal chelation reactions. The types of chemical functionalities and their position on the phenol ring modulate its antioxidant potential and the capacity to act as a radical scavenger [39, 40]. Several assays have been developed to measure antioxidant activity and the most commonly used with natural product extracts is the 1,1-diphenyl-2-picrylhydrazyl radical scavenging assay (DPPH) [41]. The DPPH assay is commercially available, inexpensive and technically simple to execute, being one of the main reasons the antioxidant activity of natural product extracts is frequently reported in the literature, and in the general media, as a source of benefits such as healthy ageing and cancer prevention. It is also probable that the antioxidant activity of phenols is largely responsible for the broad, promiscuous activity of this class of compounds reported from screening assays. In addition to antioxidant potential, *ortho*-dihydroxy aryl derivatives possess metal chelating properties, and high order polyphenols have the capacity to bind macromolecules through multiple, non-specific hydrogen bond interactions, thus causing aggregation, particularly when tested at near millimolar concentrations. These mechanisms are nearly impossible to optimise chemically to reach pharmacologically useful potency and selectivity, and they are largely responsible for the view of phenolic compounds as promiscuous, “nuisance assay positives” by the drug discovery community [42]. Nevertheless, natural phenols keep being reported as anthelmintics in the literature, and herein we discuss their nematocidal activity. Cinnamoyl derivatives, polyphenols and flavanols will be reviewed in their own subsection below.

Carvacrol and thymol (Fig. 2) are commonly found in plant extracts and have displayed activity against the model nematode *C. elegans* [43]. When treating worms *in vitro* at a concentration of 670 µM for 24 h, the two compounds caused 100% mortality, unlike p-cymene, which displayed little efficacy in this assay. Microscopy showed that thymol-treated *C. elegans* displayed burst or bent morphologies. Carvacrol and thymol were also shown to be effective against the pig roundworm, *Ascaris suum, in vitro*. Both compounds killed 80% of *A. suum* worms after treatment for 24 h at a concentration of 330 µM, while p-cymene at 370 µM killed only 20% after 24 h [43]. Thymol has also been described as an inhibitor of *H. contortus* egg hatching (IC_50_ = 2.4 mM), L1 to L3 larval development (IC_50_ = 0.83 mM), and L3 motility (IC_50_ = 3.3 mM) [44].

Carvacrol and thymol are more toxic to human cells than their acetate derivatives. It was hypothesised that the difference was due to acetylation suppressing hydroxy radical formation, and both carvacrol acetate and thymol acetate (Fig. 2) were compared with their non-acetylated parent compounds in a nematocidal study using *H. contortus*. In the carvacrol study [45], it was found that the acetate had a somewhat weaker anthelmintic activity than free carvacrol against *H. contortus in vitro* (egg hatching EC_50_ = 11.3 mM and 1.1 mM; larval development EC_50_ = 2.0 mM and 1.3 mM, respectively). Both carvacrol- and carvacrol acetate-treated adult *H. contortus* displayed morphological alterations in the cuticle and the vulvar flap, suggesting that both have the same mechanism of action. Dosed at 250 mg/kg (once), presumably p.o., carvacrol acetate reduced gastrointestinal nematode faecal egg counts in infected sheep by 66%, while its LD_10_ and LD_50_ in mice by the same route were 567 mg/kg and 1545 mg/kg, respectively. A metabolite study was not undertaken, so it was not known whether carvacrol acetate was possibly metabolised to carvacrol under the study conditions and, therefore, the real active constituent is unknown. The thymol acetate study [46] produced very similar results. Acetylation of thymol reduced its acute toxicity to mice, but it also caused a 10-fold reduction of potency in an egg-hatch assay and a slightly decreased potency in a larval development assay when compared with the parent thymol. These results suggest that the modest nematocidal activity of these phenolic molecules is likely derived from their intrinsic antioxidant character.

### Cinnamoyl derivatives and polyphenols (tannins, flavonoids and isoflavonoids)

Cinnamic acid is an intermediate in the biosynthesis of other natural phenolic compounds, such as coumarates, flavonoids and tannins (Fig. 3). Several cinnamic acid derivatives were isolated from fractions of natural extracts with anthelmintic activity. One such study found that leaves of *Acacia cochliacantha* collected in Mexico contained a high percentage of caffeic acid, ferulic acid and p-coumaric acid, and their respective esters (Fig. 3) [47]. Fractions containing these cinnamic acid-like derivatives were tested in a *H. contortus* egg-hatching inhibition assay. At a concentration of 1 mg/ml (~ 5 mM), all compounds tested displayed an egg hatch inhibition of 71–98%, in agreement with a previous field study that found that goats fed *A. cochliacantha* foliage excreted less *H. contortus* eggs than those on a different feed (cf. [47]), suggesting that the reduction in egg counts was due to the presence of cinnamic acid derivatives in this plant.

As introduced in the subsection on phenolic compounds, polyphenols (also named hydrolysable or condensed tannins) are able to bind non-specifically to macromolecules and aggregate them when the compounds are present at near millimolar concentrations, a process central to the leather tanning process (cf. [48, 49]). Condensed tannins, such as procyanidins (Fig. 3), were the primary constituents isolated from fractions of *Tilia* flowers and goat willow leaves, while prodelphinidins (Fig. 3) were the main components isolated from fractions of black and red currant leaves. These fractions were tested in an exsheathment inhibition assay using the L3 stage of the Juan strain of *H. contortus* [50] and compared with the activities of the flavonoids naringenin, quercetin and luteolin (Fig. 3). In this assay, the prodelphinidin-rich fractions showed similar activity to the flavonoids (IC_50_ = 60–90 µg/ml; ~ 100–150 µM) and were 2-fold more potent than the procyanidin-rich fractions (IC_50_ = 140–176 µg/ml; ~ 250–300 µM). Luteolin was also found to possess anthelmintic activity against *Trichuris muris* (IC_50_ = 9.7 µg/ml; ~ 34 µM) (and against *S. mansoni*, not reviewed here) [51]. Electron microscopy revealed that luteolin-induced cuticular or tegumental damage in all worms tested.

A series of 33 hydrolysable tannins were isolated from cold-acetone plant extracts from Finland and evaluated against *H. contortus*, measuring inhibition of egg hatching and motility of L1 and L2 larvae [52]. Of the tannins evaluated, pentagalloylglucose, tellimagrandin II and hippophaenin B were the most potent at reducing egg hatching (> 50% inhibition at 0.5 mM), while pentagalloylglucose, heptagalloylglucose, tellimagrandin II and casuarinin (Fig. 3) all had an IC_50_ of < 0.5 mM when inhibiting the motility of L1 and L2 larvae. Perhaps worryingly, cryo-scanning electron microscopy showed that pentagalloylglucose formed aggregates on the surface of the L1 and L2 larvae and eggs, profoundly affecting the buccal capsule and the anterior amphidial channels. This suggests that its anthelmintic effects may be due to tannin deposition on biological surfaces, rather than to interactions with specific targets. The degradation of pentagalloylglucose and casuarinin was also measured following incubation in phosphate-buffered saline (PBS) or in ‘egg matrix’ solution, and it showed considerable hydrolysis of the gallic acid residues plus oxidation. The authors determined that there was an optimal molecular weight for activity (940 Da) and used this parameter, together with the number and linkage type of gallic acid residues, to propose an empirical equation predicting the anthelmintic potency of hydrolysable tannins [52].

Gallic acid analogues, the ellagic and gentisic acids, were isolated from the axlewood tree, *Anogeissus leiocarpus* [53]. Ellagic acid exhibited the greatest activity against the free-living nematode *C. elegans* and the bovine filarial nematode *Onchocerca ochengi*, with an IC_50_ = 90 µM against the adult and an IC_50_ = 30 µM against the microfilariae of *O. ochengi*. The potency of ellagic acid against wildtype *C. elegans* (IC_50_ = 85 µM) was unchanged in strains resistant to levamisole and ivermectin. However, this compound displayed 10-fold less activity against the CB3474 strain, resistant to albendazole, suggesting that ellagic acid activity is affected by some albendazole resistance mechanisms. On the positive side, ellagic acid showed a good safety profile in rats and, therefore, may represent a reasonable starting point for development as an anthelmintic agent. The multiple hydroxyl groups are, however, frequent sites of metabolism and would probably need to be chemically protected.

Other gallic acid analogues were isolated from the fruits of *Acacia nilotica* from the northern areas of Cameroon [54]. (+)-catechin-3-O-gallate, and four related proanthocyanidins: (+)-epicatechin-3-O-gallate, (+)-gallocatechin, (−)-epigallocatechin and (−)-epigallocatechin-3-O-gallate were all isolated and evaluated against *C. elegans* and *O. ochengi*. All derivatives displayed IC_50_ = 1–11 µM against adult and microfilariae of *O. ochengi*, a reasonable potency, even though they were all less active against wildtype *C. elegans* (IC_50_ = 34–350 µM). Importantly, *C. elegans* strains resistant to albendazole, levamisole or ivermectin were equally susceptible to these compounds, implying that the gallic acid analogues tested were not susceptible to resistance mechanisms affecting these commercial anthelmintics. However, all derivatives also displayed toxicity against a human cell line (Caco-2) at concentrations comparable with their potency against *C. elegans* (see [53]). This raises questions about the pharmacological developability of this set of proanthocyanins.

**Fig. 4:**
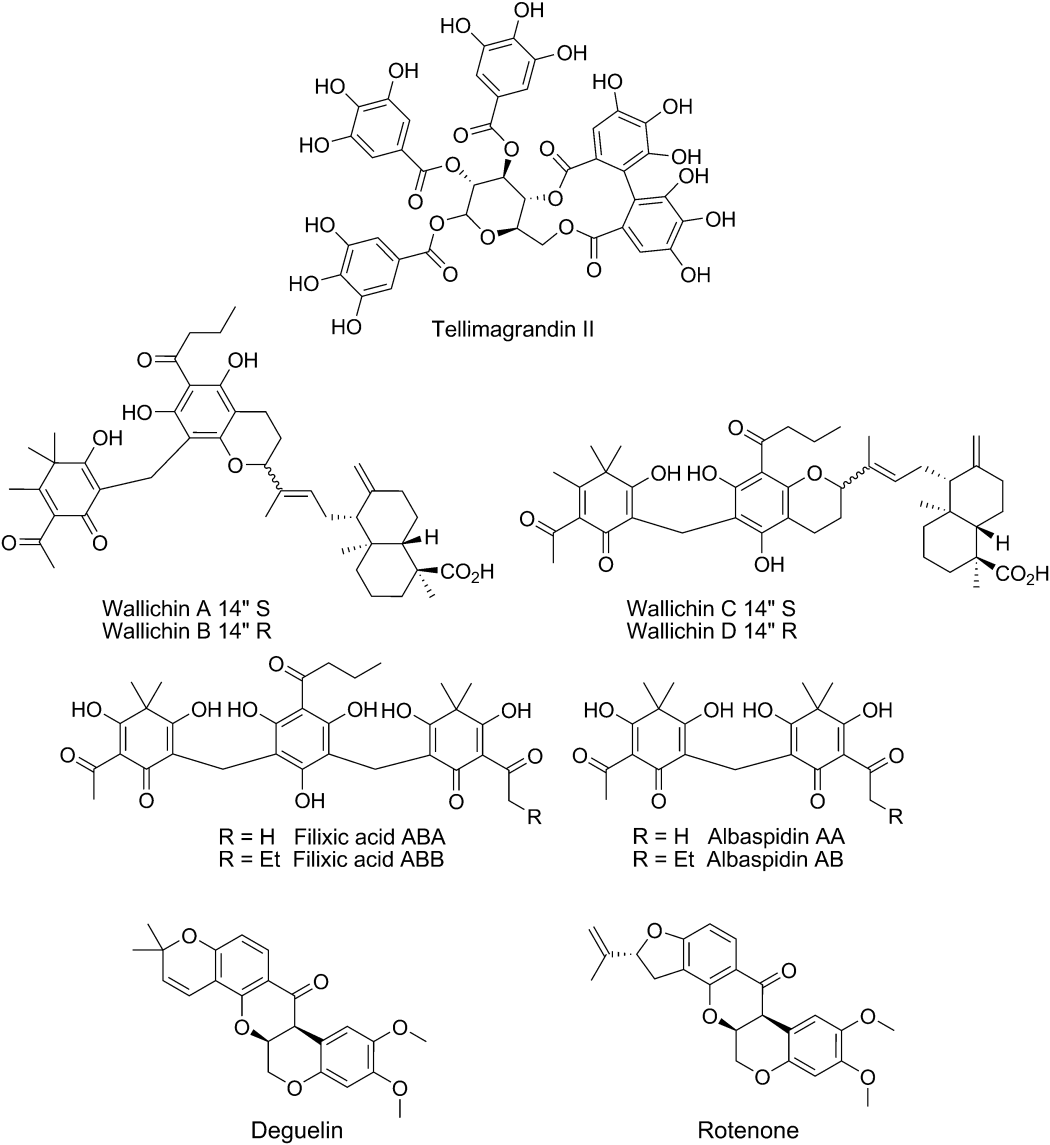
Polyphenols (cont.) (tannins, flavonoids and isoflavonoids)

Polyphenols are also found modified with other natural synthons. Socolsky et al. [55] isolated several unusual terpenylated acylphloroglucinols, wallichin A-D, filixic acids ABA and ABB, and albaspidin AA and AB (Fig. 4), from the rhizomes and scales of the fern *Dryopteris wallichiana*, traditionally used to treat helminthiases. The authors then evaluated these derivatives in a viability assay against the L4 larval stage of the rat nematode *Nippostrongylus brasiliensis.* It was found that wallichins A and B as well as flilixic acids displayed higher potency than unmodified polyphenols, with LD_50_ = 22–37 µM, while wallichin C and the albaspidins displayed 5-fold weaker activity. The authors hypothesised that a free hydroxy group at the C-4′ position of wallichins A and B was important for modulating activity.

The isoflavonoids deguelin and rotenone (Fig. 4) were discovered from a screen of a small natural product library against exsheathed third-stage (xL3) larvae of *H. contortus* [56]. The two compounds are commonly isolated from plants of the family Leguminosae [57] and have been reported previously to display a range of activities, such as anti-inflammatory [58], apoptotic [59] and insecticidal [60]. However, rotenone exhibits potent mammalian cytotoxicity [61, 62] and, therefore, its nematocidal activity was not further investigated. Deguelin was relatively benign to mammalian cells in the screening study (neonatal foreskin fibroblast cells; IC_50_ > 50 µM), while showing an IC_50_ = 14 µM against xL3 and an IC_50_ = 0.004 µM against L4 in a 72-h motility assay. Deguelin also blocked development of xL3s to the L4 stage after 7 days, with an IC_50_ = 3.2 µM [56]. Transcriptomic responses of xL3 *H. contortus* to deguelin treatment identified differential gene transcription associated with energy metabolism, particularly oxidative phosphorylation and mito-ribosomal protein production, supporting an inhibition of the respiratory complex I as the mode of anthelmintic action, as also proposed for the compound’s effects on mammalian cells [63]. In mammals, complex I inhibition by deguelin seems to act in parallel to the disruption of the PI3K-Akt kinase signalling pathway [64], which has been linked to neurotoxicity [63, 65]. Further analyses are needed to determine whether deguelin targets the same pathways in mammals and nematodes, which would enable a better understanding as to whether it is possible to refine the selectivity of this potent natural nematocide.

### Terpenes

From a formal point of view, terpenes are the products of condensation of at least two isoprene monomers (as in monoterpenes), and, more frequently, three (sesquiterpenes), four (diterpenes) or six (triterpenes) five-carbon units. Terpenoids (=isoprenoids) are terpenes modified by the addition of heteroatoms, frequently oxygen. Chemical formalisms aside, there are two main routes of terpene and terpenoid biosynthesis. Many organisms produce them utilising mevalonic acid as a key intermediate (the mevalonate pathway). An alternative, non-mevalonate pathway results in the production of isopentenyl pyrophosphate and dimethylallyl pyrophosphate, which are key synthons for the production of higher order terpenes, such as the prenyl derivatives and triterpenoids discussed in their own subsections below. Terpenes are susceptible to oxidation by cytochrome P450 enzymes, a main route for the production of oxygenated terpenoids [66]. Oxidation events include the formation of lactones, sesquiterpene quinones and hydroxyl quinones and epoxides, all of them reactive species that can endow this chemical class with biological activity in the correct molecular environment.

**Fig. 5:**
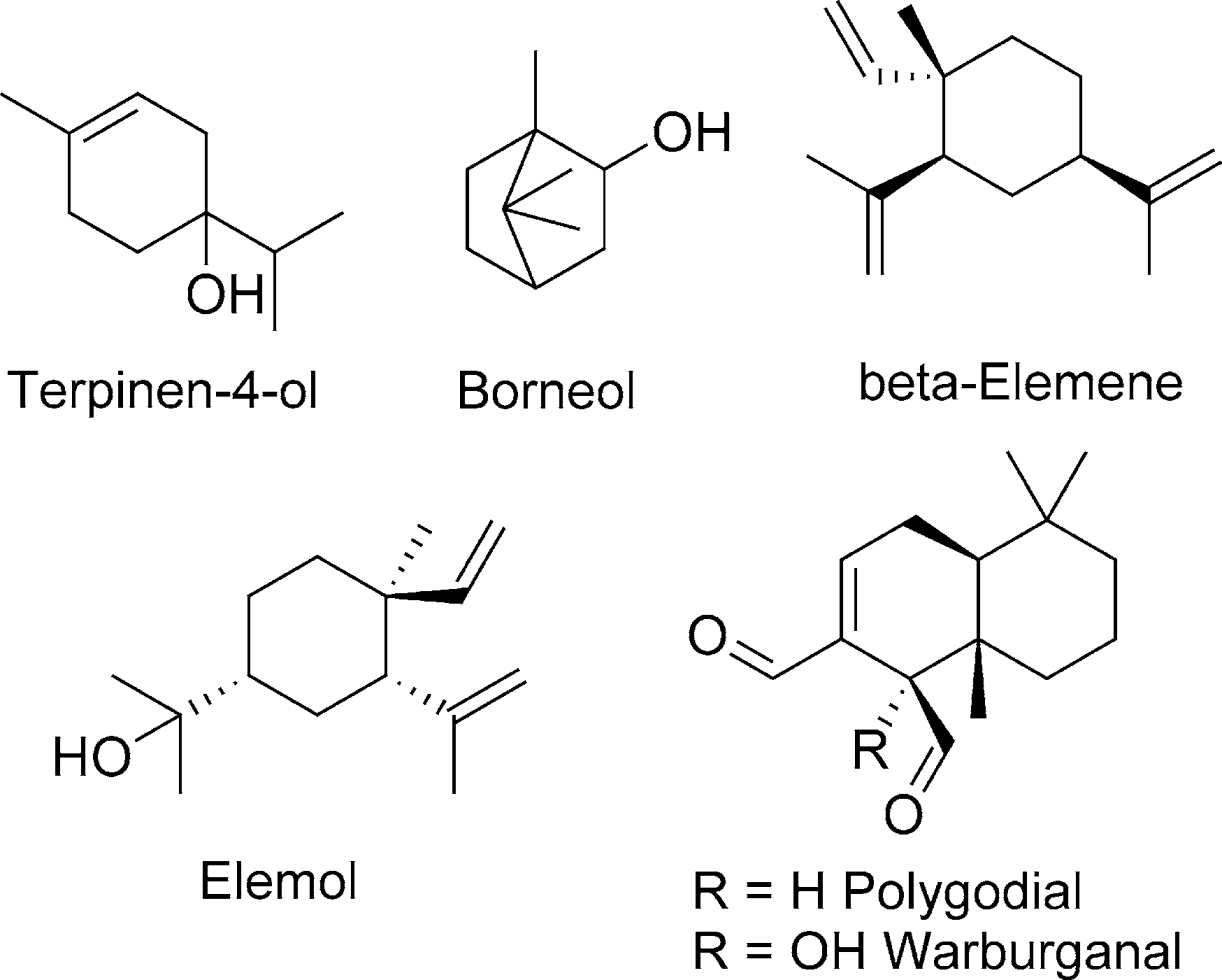
Terpenes (terpenoids or isoprenoids)

In recent literature, several terpenoid natural products were found to possess nematocidal activity. Terpinen-4-ol (Fig. 5), commonly isolated from conifers and *Melaleuca alternifolia*, the source of tea tree oil, is well known for its modest antimicrobial activity [67]. A recent study [68] also demonstrated some activity of terpinen-4-ol against *H. contortus*. In this study, in which a “nanostructured” formulation of the oil was also tested, terpinen-4-ol was the most active test article, with an IC_50_ = 4.1 mM in the egg hatch inhibition assay, and an 82% inhibition of L3 migration at 22 mM. These *in vitro* potencies were too low to consider *in vivo* testing in animals.

A prickly shrub found in China and Taiwan and used in traditional medicine, *Zanthoxylum simulans*, produces an essential oil containing a variety of alkaloids, coumarins and flavonoids, including a significant amount of the terpenes borneol and β-elemene (Fig. 5). The two terpenes were evaluated against larval stages of *H. contortus* [69]. Borneol inhibited *H. contortus* egg hatching (IC_50_ = 9.7 mM) and larval development (IC_50_ = 12.9 mM), and at 130 mM it suppressed larval migration by 98%, while β-elemene was poorly active or completely inactive in all assays, despite the high millimolar concentrations used; these results essentially led to the elimination of these terpenoids as anthelmintic starting points.

Warburganal and polygodial are structurally related sesquiterpenes containing two aldehyde moieties (Fig. 5). The two compounds were isolated from the medicinal plant *Warburgia ugandensis*, native to eastern and southern Africa [70]. An ethanolic plant extract was fractionated, guided by the detection of anthelmintics activity against *C. elegans*, and warburganal and polygodial were recovered among the most active natural constituents, with respective IC_50_ values of 28.2 µM and 13.1 µM. The two sesquiterpenes were equally active against several drug resistant strains of *C. elegans*, suggesting that the compounds were not susceptible to drug resistance mechanisms affecting commonly used anthelmintics. Several analogues of the two sesquiterpenes were obtained and evaluated for preliminary structure-activity relationship studies. It was found that at least one of the two aldehyde groups present in these structures was required for activity, suggesting that these chemically-reactive motifs contribute to the observed anthelmintic effect. Mechanism of action studies were also undertaken, based on similar studies of polygodial using the yeast *Saccharomyces cerevisiae* and mammalian cells, where this compound was found to act as an uncoupler of mitochondrial oxidative phosphorylation [71]. In *C. elegans,* polygodial was found to inhibit mitochondrial ATP synthesis with an IC_50_ = 1.8 µM and a phenotype consistent with inhibition of mitochondrial ATP synthesis. Collectively, these data suggest that polygodial is a worthwhile compound to be considered for further investigation as a nematocide. However, selectivity will need to be addressed, since polygodial inhibits mammalian ATP synthesis to a similar degree as in *C. elegans* (see [71]).

### Prenyl derivatives

**Fig. 6:**
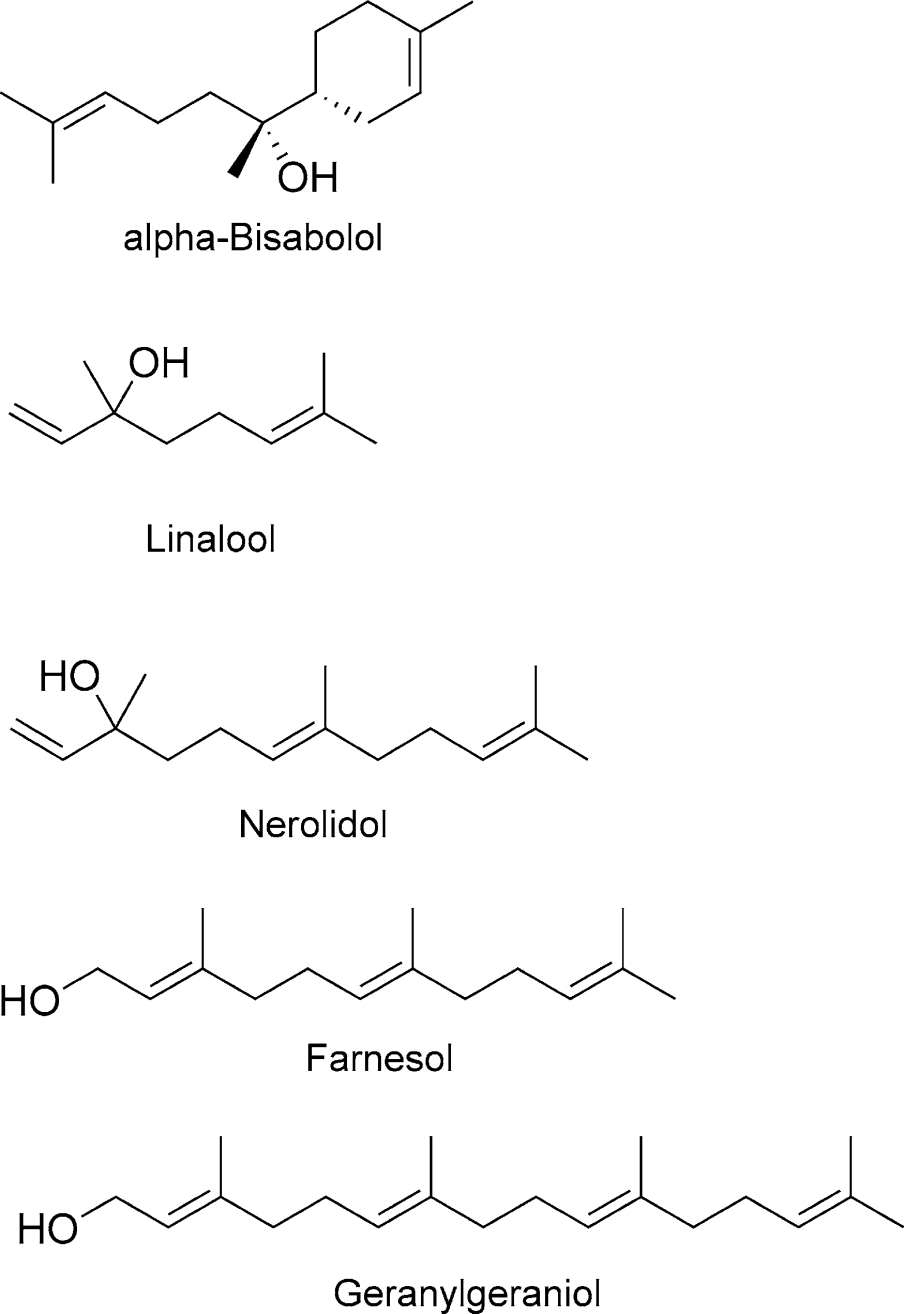
Prenyl derivatives (linear terpenes and terpenoids)

The final biosynthetic products from the non-mevalonate pathway of isoprenoid biosynthesis, geranyl pyrophosphate and farnesyl pyrophosphate, are precursors to linear mono-, di- and sesquiterpenes, called prenyl groups, which are frequently isolated as natural products with some additional modifications. The monoterpene alcohol linalool (Fig. 6), present in hundreds of plant species, exists as a mixture of two enantiomers to which numerous biological activities have been ascribed, including significant toxicity against transformed cells (cf. [72]). Linalool, presumably the racemate, has recently been put forward as a chemical starting point for anthelmintic drug development, along with other natural products [73]. The compounds in this study were selected from phytochemical databases and filtered chemoinformatically for favourable ADME and drug-like properties. Compounds were further screened computationally for binding to a modelled structure of glutathione S-transferase (GST) from the filarial worm *Brugia malayi*, and the best hits were biochemically tested at 1 µg/ml against a crude GST preparation from the heartworm *Dirofilaria immitis* (due to a lack of access to *B. malayi*). Linalool inhibited DiGST by 98% at 1 µg/ml (~ 6.5 µM) in this assay, and it was thereby assumed to possess anthelmintic efficacy, although this was not directly tested, a problem when interpreting the results. Linalool is sensitive to an extensive metabolism that generates multiple derivatives with biological activity [72] and its purported biological target in nematodes and insects, GST, is usually present as a family of multiple isoenzymes in metazoans; the *H.* *contortus* genome, for example, has at least 8 distinct genes encoding GST isozymes (ParaSite@WormBase; release 11) [74]. Presently, it is unknown whether any of these enzymes is singularly essential and thus represents a good anthelmintic target, or how many functionally redundant isozymes can be simultaneously inhibited to a sufficient extent by a single compound. These are some of the biological and chemical questions that need to be addressed ahead of a drug development effort with these compounds.

Three prenyl alcohols present in the essential oil from *Matricaria chamomilla* and multiple other plants were described as active *in vitro* and *in vivo* against L3 larvae of *Anisakis simplex* [75, 76]. Compounds were tested *in vitro* at three different concentrations and scored as active down to 15.6 µg/ml (70 µM) for farnesol and nerolidol (presumably a racemate in the case of nerolidol) and down to 32.5 µg/ml (146 µM) for α-bisabolol (Fig. 6). At those concentrations, these compounds arrested motility, abolished infectivity of treated *Anisakis* larvae in rats and caused extensive tissue damage to the worms. *In vivo*, they protected rats from *Anisakis*-induced gastric lesions when dosed at ~ 300 mg/kg simultaneously with infection, although larval mortality *in vivo* was lower with the pure compounds than that achieved when using complete plant essential oil [75, 76], suggesting the existence of beneficial synergistic interactions within the unfractionated extract. To a similar or even greater extent than linalool, these prenyl alcohols are all very hydrophobic compounds, with chemical structures prone to being extensively metabolised and with numerous known biological activities, although always with relatively low potency [77, 78].

The activity of these and other chemically related prenyl alcohols has been confirmed in *C. elegans* (see [79]), albeit with the same low potencies (IC_50_ = 50–100 μM). Activity against this free-living nematode facilitates, in principle, the use of genetic and biochemical techniques to identify the mode of anthelmintic action. However, low potencies may make these expectations difficult to achieve in practice, because high compound concentrations will be needed to observe unambiguous results, and this creates problems of compound solubility and non-specific effects.

Despite the low chemical developability of prenyl compounds, their biology makes them enticing scaffolds for the generation of derivatives able to disrupt essential pathways in target organisms. Aliphatic prenyl derivatives are signalling molecules in multiple cellular pathways and old models of them acting by simply “sticking” to lipid bilayers are giving way to observations of specific interactions with cognate protein domains [80]. Prenyl compounds can affect cells by interfering with the synthesis or transfer of essential farnesyl and geranylgeranyl groups to proteins [81]. Their signalling roles are also worth considering when thinking of anthelmintic analogues. It is noteworthy in this context that insect juvenile hormone is a linear sesquiterpene, and, although not yet isolated from other Ecdysozoa, this hormone and related farnesol esters interfere with ecdysis in nematodes at relatively high micromolar concentrations [82]. This suggests that it may be possible to usefully disrupt endocrine signalling in nematodes if a more potent and water-soluble prenyl mimic can be found.

### Triterpenoids and triterpenoid glycosides (saponins)

Six studies discussed below reported triterpenoid compounds with some measure of inhibitory activity against animal parasitic nematodes. Triterpenoids constitute a large class of natural lipids synthesised from a 30-carbon precursor, modified by addition of heteroatoms (generally oxygen) and cyclizations that commonly generate tetracyclic (e.g. steroids) or pentacyclic (e.g. oleanoates) structures. Their conjugation to sugar residues produces amphiphilic glycosides, termed saponins, present in most plant species and displaying the surfactant (detergent) properties expected from their chemical structure [83]. The physicochemical properties of saponins allow them to disrupt molecular associations that depend on hydrophobic interactions (although it is apparently their bitter taste that deters herbivores). Triterpenoids have large hydrophobic surfaces able to bind, with biologically relevant affinity, to hydrophobic patches in proteins, sterol-rich membrane domains and binding sites for steroid or isoprenoid ligands. Therefore, they are promiscuous binders, particularly at the relatively high concentrations required for anti-infective use and, consequently, triterpenoids have been linked to numerous biological activities [84–88]. While saponins are water-soluble, and generally toxic, the unglycosylated free triterpenoids tend to be less toxic but are challenging to develop into oral drugs, mainly because of their low aqueous solubility. This difficulty has been overcome with sophisticated formulations for price-insensitive applications [89].

**Fig. 7:**
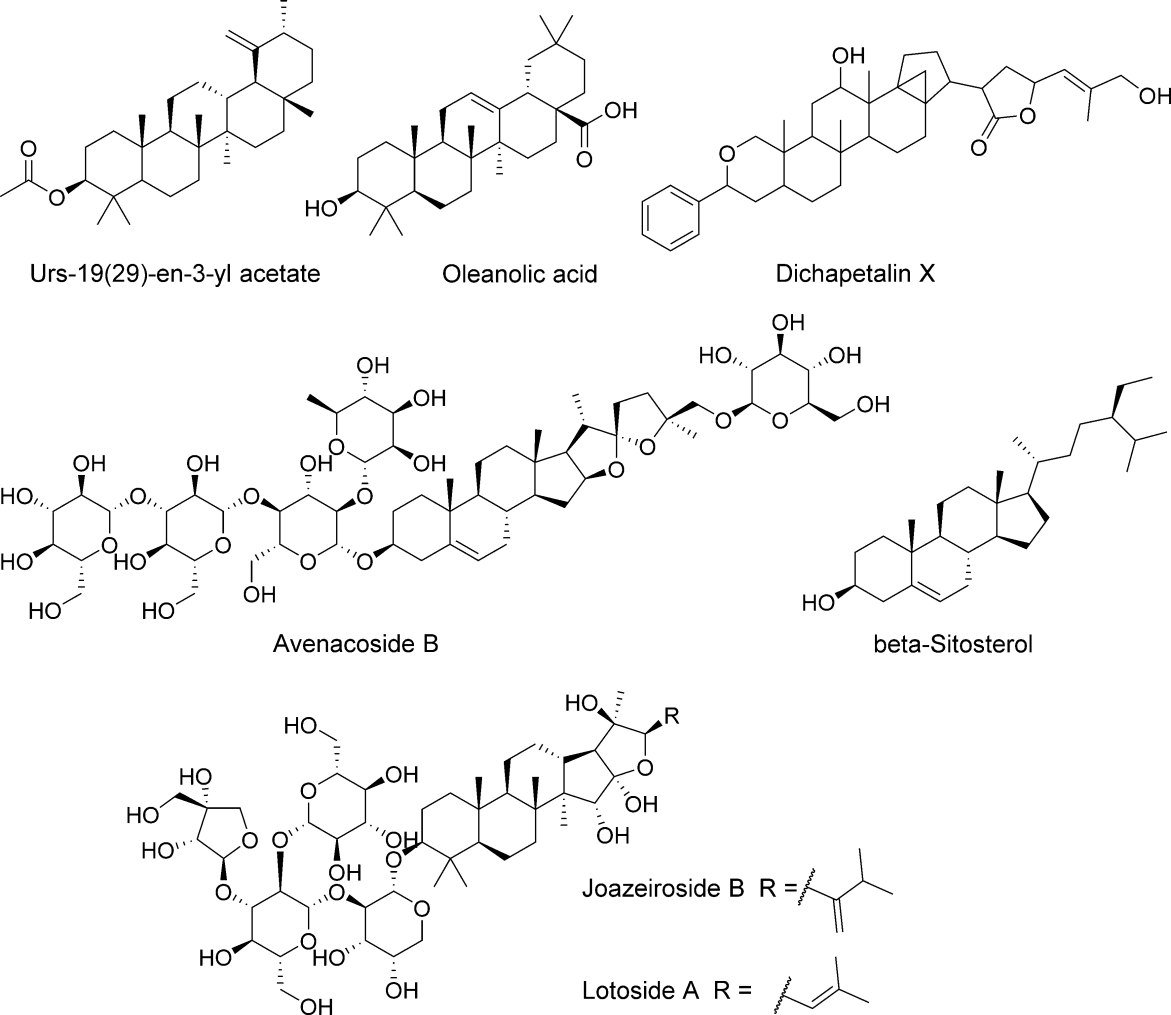
Triterpenoids and triterpenoid glycosides (saponins)

Recent reports on triterpenoids active against parasitic nematodes described compounds extracted from four different tropical plants, a common crop (oats) and an edible mushroom. An ethyl acetate extract from the latex of the shrub *Calotropis procera* (Ait.) was shown to have activity against *H. contortus*, with a reasonably potent EC_50_ against egg-hatching (1.6 mg/ml), larval development (0.22 mg/ml) and adult motility (~ 0.05 mg/ml) [90]. Three major components were identified upon fractionation of the crude extract: urs-19(29)-en-3-yl acetate (Fig. 7), (3β)-Urs-19(29)-en-3-ol and 1-(2′,5′-dimethoxyphenyl)-glycerol. Surprisingly, even though they were obtained in pure form, no individual activities were reported, and it is thus unknown at this stage whether the anthelmintic properties of the extract can be attributed to either of the three compounds, to their combination or, less probably, to a minor component of *Calotropis* spp. latex that was not considered in the study.

The saponin fraction extracted from the bark of *Zizyphus joazeiro*, a shrub or small tree native to Brazil, was shown to have activity against caprine gastrointestinal nematodes (> 90% *Haemonchus*, with *Oesophagostomum* and *Trichostrongylus* species making up the rest) [91]. Intriguingly, the preparation displayed ovicidal activity with promising potency (EC_50_ = 1.3 mg/ml), but no activity against L3 larvae. This could be mainly due to permeability differences between egg-shell and larval cuticle, as the extract seemed to possess clear cytotoxic activity, with IC_50_ = 0.2–0.3 mg/ml against Vero cells in culture. Joazeiroside B and lotoside A (Fig. 7) were identified among the saponins in the preparation, an identification supported by an earlier report of their presence in this plant species [92]. The authors proposed that joazeiroside B and lotoside A are responsible for most of the observed activity.

Saponin cytotoxicity was also encountered when analysing active methanolic extracts from seeds, bark and leaves from the Sri Lankan tree *Dipterocarpus zeylanicus*. All extracts displayed activity against the adult and microfilarial stages of the bovine filarial nematode *Setaria digitata* [93]. Considering toxicity to human cells, the extract from seeds seemed to have better selectivity (although no more than 2-fold) than extracts from other plant parts, and it was fractionated to identify its active components. Four pure compounds, representing ~ 0.4% by weight of the total methanolic extract, were identified and studied. The two most abundant and potent, although also cytotoxic, were the 3-glucosyl and 3-arabinosyl saponins of oleanolic acid (Fig. 7). Two minor components also identified were free triterpenoids: a *regio*-isomer of oleanolate and betulinic acid, both found to be inactive in this study. Given the multiple biological activities attributed to oleanolic acid (cf. [94]), it was considered interesting to hydrolyse the saponins and test the released oleanolate. Senathilake et al. [93] measured an EC_50_ of ~ 40 µM for inhibition of motility by oleanolic acid, both in microfilariae and adult worms, with selectivity indices > 10-fold relative to cytotoxicity. At concentrations of more than twice the EC_50_, signs of tissue damage in worms were observed, with apoptotic bodies, DNA fragmentation and increased levels of oxidative stress markers, concordant with previously described effects of oleanolate in rapidly growing cells. Interestingly, a search for the free terpenoid in *Dipterocarpus* seeds failed, and, thus, the basis for the higher selectivity of the seed extract relative to those from other plant parts remained unexplained.

Different terpenoids were detected in organic solvent extracts from roots of the West African plant *Dichapetalum filicaule*, which had ovicidal activity against the human hookworm *Necator americanus* [95]. Silica gel column-fractionation and analysis by NMR and MS revealed the presence of numerous triterpenoids, and the nematocidal activity of two of them, dichapetalins A and X (Fig. 7), was investigated. Hatching of hookworm eggs was inhibited by both compounds, with an EC_50_ of 162 µg/ml and 523 µg/ml for the A and X forms, equivalent to 277 µM and 745 µM, respectively. Other triterpenoids detected in the extract, such as pomolic acid, have been described previously to have multiple biological activities, but they were not tested in the assay.

A more sustainable plant source than those referred to above is oats (*Avena sativa*); the saponins avenacosides A and B (Fig. 7) were extracted from seedlings using methanol [96]. The authors also sought to emulate the plant response to fungal infection by treating the saponins with avenacosidase, a β-glucosidase that produces the 26-desglucosyl form of the compounds. The products of the enzymatic treatment still retain their glycolipid character due to a tetrasaccharide at position 3 of the triterpenoid core, but the removal of glucose at position 26 reportedly makes them effective disruptors of fungal membranes [97]. Given the use of oats as a traditional remedy for humans and cattle, avenacoside B and its 26-desglucosyl form were each tested against fungi, as well as against eggs and larvae of the parasitic nematode *Heligmosomoides bakeri*. Although growth of the fungus *Trichoderma harzianum* was inhibited *in vitro*, anthelmintic activity was apparently confounded by the presence of 2% ethanol in the preparations, which claimed much of the effect observed relative to medium-only controls in most tests, perhaps with the exception of Rho-123 dye accumulation, which would indicate an interference with a P-glycoprotein-type efflux pump [96].

The only non-plant source of an anthelmintic triterpenoid reported since 2010 was the edible fungus *Pleurotus djamor*. A hydro-ethanolic extract of fruiting bodies (mushrooms) was fractionated by direct phase silica gel chromatography, and one fraction with ovicidal activity against *H. contortus* was analysed by gas chromatography-mass spectrometry (GC–MS). It was found to contain mostly free fatty acids plus ≤ 1% of the triterpenoid β-sitosterol (Fig. 7), as determined by ion counts [98]. It is, however, to be expected that the abundant free fatty acids present in the most active fraction were responsible for the largest share of the extract’s ovicidal activity, given that their concentrations greatly exceeded the 241 µM of β-sitosterol present.

In summary, no new nematocidal triterpenoids were reported in the reviewed literature, and the reported ones were previously known and displayed relatively low potencies and narrow selectivity windows in anthelmintic assays, making their development as nematocidal compounds challenging. Nevertheless, it is possible to develop triterpenoids into anti-infective drugs (e.g. fusidic acid), and nematodes are known to use steroid-hormone signalling (cf. [99]), which could potentially be disrupted to obtain an anthelmintic effect if developable analogues can be identified.

### Macrocycles

Studies have described anti-nematode activities of macrocyclic lactones or related compounds. Macrocycles are a large class of bioactive natural products that includes the macrolide and glycolipopeptide antibacterials and the cyclosporin and rapamycin immunosuppressants. Nematocidal macrocycles (avermectins and their aglycons, the milbemycins) are produced by several *Streptomyces* species and were discovered in the 1970s [100]. The discovery and development of the avermectins were awarded the 2015 Nobel Prize in Physiology or Medicine, rewarding the large contribution of a semi-synthetic derivative, ivermectin, to the control of lymphatic filariasis and river blindness. Although not compliant with several drug-likeness rules, avermectins are amongst the most potent pesticides in use, with efficacious doses against nematode and arthropod infections well below 1 mg/kg, even as a single administration (reviewed in [101]). Their recognised mode of action is the activation of a neuronal glutamate-gated chloride ion channel [102].

**Fig. 8:**
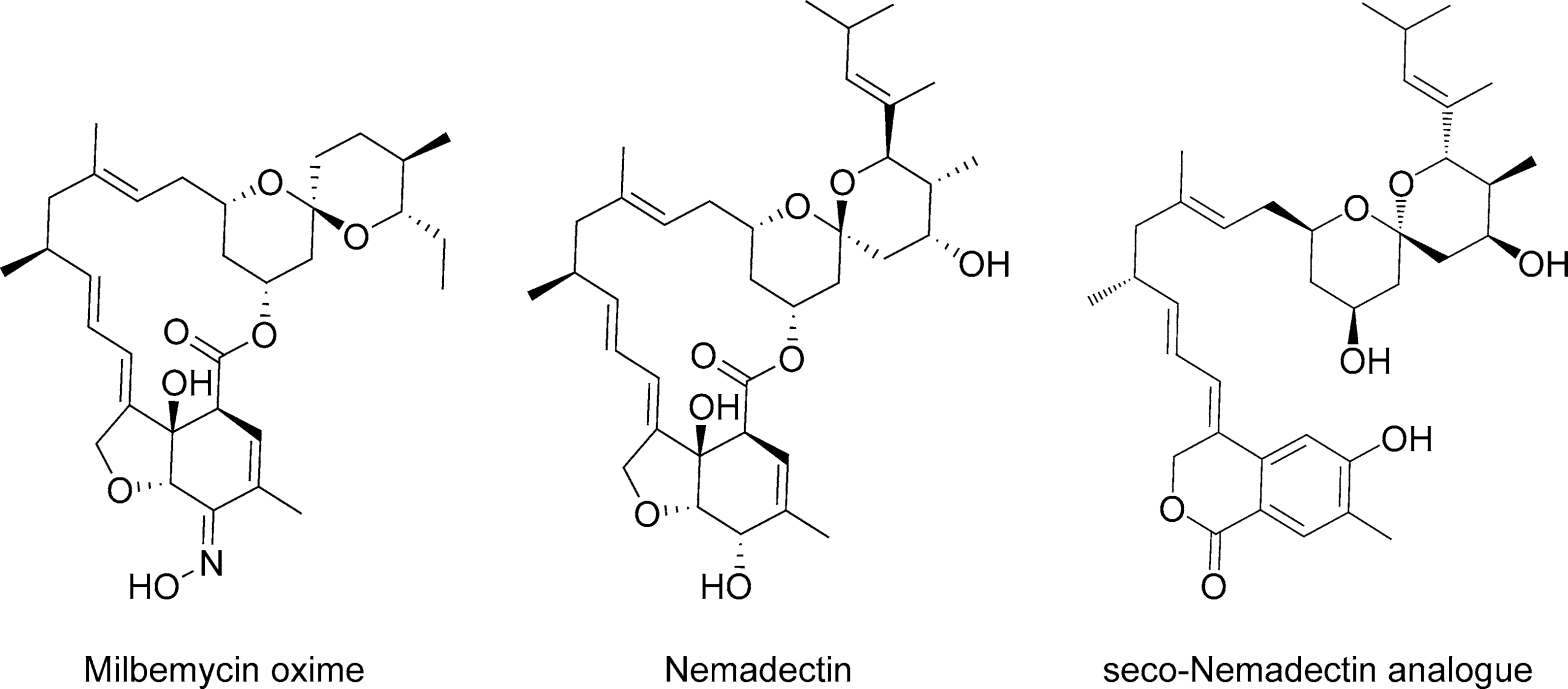
Macrocycles and analogues

More recently, Xiang et al. [103] extracted mycelium from *Streptomyces microflavus* with methanol and fractionated the resultant product by chromatography on silica gel, thus purifying a compound active against *C. elegans* and adult two-spotted spider mites (*Tetranychus urticae*). *In vitro* potency was surprisingly high (EC_50_ = 15.4 µg/ml or 26 µM against *C. elegans*), taking into account that, although the compound shares structural motifs with the macrocyclic lactone nemadectin (a milbemycin), it lacks the lactone ring itself (Fig. 8). Activity was confirmed with four similarly open structures obtained from *Streptomyces bingchenggensis*, showing that these much more flexible compounds possess biological activity found previously only on the cyclic milbemycins [103]. It is not known at this early stage whether the new compounds share the mode of action of the closed macrocycles and whether they possess pharmacological advantages or disadvantages. It would be especially interesting to learn whether they also utilise the unexpected route of entry into nematodes recently described for ivermectin [104].

One study on macrocyclic anthelmintics confirmed the clinical efficacy of the semi-synthetic oxime of a milbemycin (Fig. 8) in treating dogs infected with *Toxocara canis, Toxascaris leonina*, *Trichuris vulpis* or *Ancylostoma caninum* [105]. Milbemycin oxime and another semisynthetic milbemycin, moxidectin, are part of numerous pesticide combinations used in animals. The latter has also recently received FDA approval for use in humans in the treatment of river blindness [106].

Although new nematocidal macrocycles have not been reported between 2010 and 2018, related open analogues that expand the possibilities for semi-synthesis, and that can be readily obtained from culturable bacteria, may contribute to the development of future commercial drugs. These novel analogues will be particularly interesting if their targets and entry routes are distinct from those utilised by the macrocyclic lactone anthelmintics, thus avoiding cross-resistance.

### Miscellaneous compounds

**Fig. 9:**
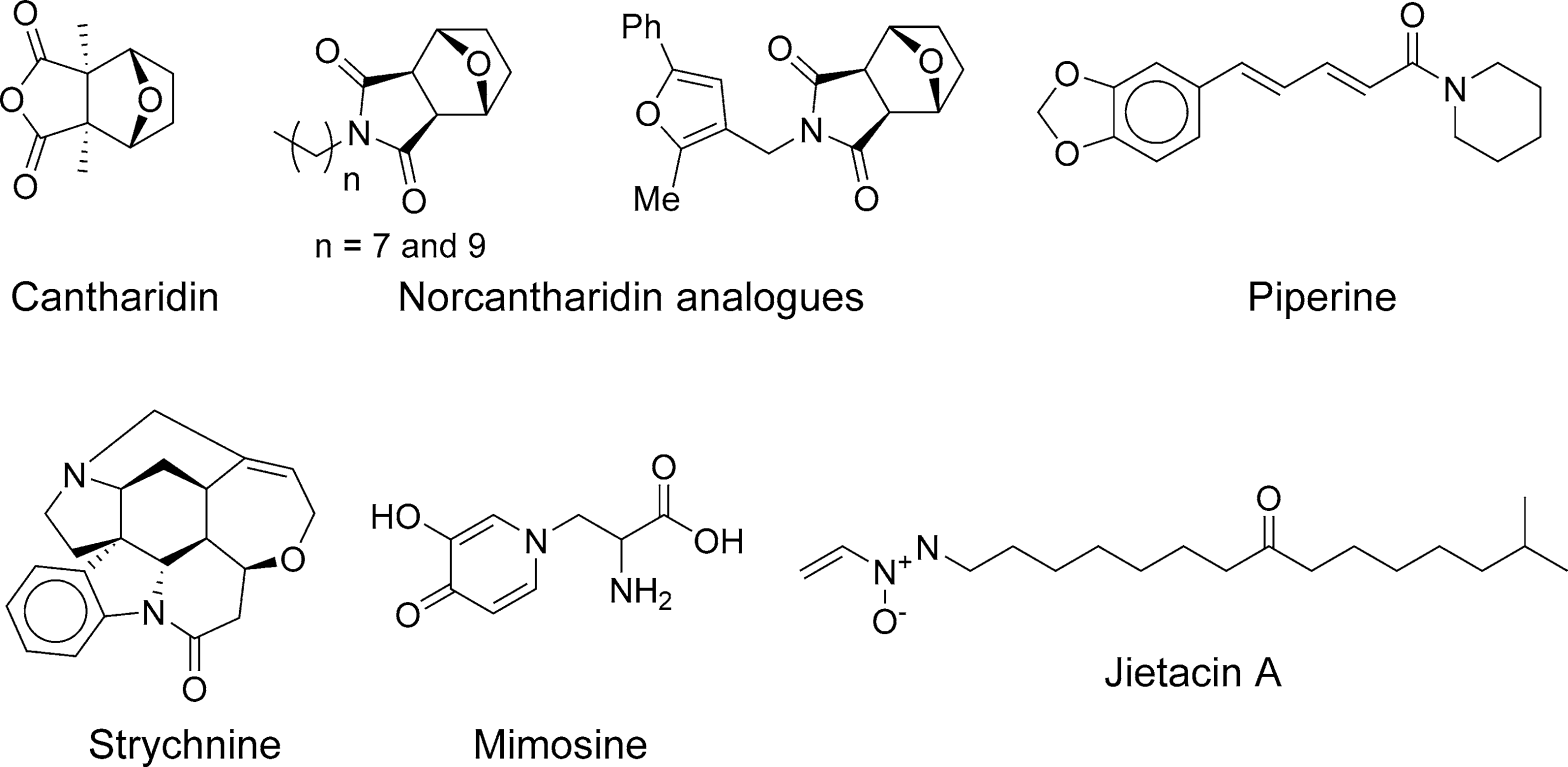
Miscellaneous compounds

The vesicant agent cantharidin (Fig. 9) from beetles of the family Meloidea has been chemically synthesised and used as an anti-cancer chemotherapy for many years [107]. Its highest affinity molecular target in humans was identified as protein phosphatase 2A (PP2A) [108]. In nematodes, some protein phosphatases are among the gene products with sex-specific expression [109–111]. This may be in apparent agreement with the purported aphrodisiac properties of cantharidin in the folk remedy “Spanish fly”, but more importantly, it has brought attention to protein phosphatases as tractable nematocidal drug targets [112]. Fifty-four analogues of the less chemically reactive norcantharidin (Fig. 9), belonging to three different chemical families, were made and tested for inhibition of *H. contortus* larval development, expecting to see a correlation between anthelmintic potency and biochemical inhibition of PP2A [112]. Unfortunately, no such correlation was observed, suggesting that chemical modification had optimised whole-animal activity by acquiring a new and unknown target. This is a relatively common occurrence in anti-infective research that confounds structure–activity relationships (SARs).

In the same study that proposed linalool as a nematocidal drug starting point (see subsection on prenyl derivatives) [73], the alkaloids piperine (from *Piper nigrum* and other *Piper* species) (Fig. 9) and strychnine (from *Strychnos nux-vomica*) (Fig. 9) were also put forward. As described for linalool, these alkaloids were also chemoinformatically selected as possible GST inhibitors and inferred to have anthelmintic activity. Piperine and strychnine were found to inhibit a crude preparation of DiGST by 80% and 87% at 1 µg/ml (3.5 µM and 3 µM, respectively). Piperine, responsible for the pungency of pepper seeds, is a known low-potency inhibitor of multiple targets [113, 114], possibly because of its chemically reactive α,β-unsaturated group. Strychnine is a known potent non-selective poison and one of the time-honoured challenges in synthetic chemistry. The known properties of the two compounds indicate that it will be difficult to develop potent and selective analogues devoid of activity against non-target organisms. Additionally, there is the problem of the multiple GST isoforms in metazoans, assuming that the enzyme is indeed their lethal anthelmintic target.

Another promiscuous molecule, a non-protein l-α-amino acid from *Mimosa pudica* and *Leucaena glauca* (mimosine) (Fig. 9), has been described previously to possess anti-cancer, anti-viral and even herbicidal properties, perhaps related to its ability to chelate metal ions (cf. [115]), a mechanism that is hard to optimise for selectivity. A complex inference was made that since phosphoramidothionates of some l-amino alcohols have insecticidal activity, and acetylcholinesterase and tyrosinase can be taken to be good insecticidal targets, phosphoramidothionates of mimosinol should be prepared and tested as inhibitors of both enzymes and as insecticides or nematocides [116]. The results were disappointing, in that activity against *C. elegans* was higher in the parent compound, mimosine (reported IC_50_ = 17 µM), than in any of its derivatives, including bis-deuterated analogues that displayed potency differences in several assays, an unexpected result which was not discussed in the report [116].

Closing this subsection, the SAR of the hydrophobic azoxy compounds jietacins (Fig. 9) has been explored and reported recently [117]. This compound class, produced by *Streptomyces* bacteria, and its nematocidal activity were first described decades ago by workers at the Kitasato Institute [118], but no reports of chemical development had appeared until 2018. Interestingly, a Japanese patent application was filed a year before the first communication by the original authors (JP S6366155 A), but it seems to have been abandoned. However, a Chinese institution did obtain local intellectual property (IP) protection for methods to prepare the compounds in 1995 (CN 1027905 C). The SAR study now published by Sugawara et al. [117] indicates that a vinyl azoxy group, located at the end of a linear aliphatic chain of 9–14 carbon atoms, is most efficacious *in vitro* and *in vivo*. Compounds were tested *in vitro* for inhibition of motility of xL3 larvae of both *Cooperia curticei* and *H. contortus*, and for inhibition of enzyme secretion by adult *Nippostrongylus brasilensis*. The most active compounds exhibited complete inhibition in the three assays at concentrations of 1–5 µg/ml (3–16 µM) and showed *in vivo* efficacy in mice infected with *Heligmosomoides polygyrus* at 25–100 mg/kg, p.o., q.d., for 4 days [117]. The parent compound, jietacin A, was also efficacious *in vivo* under the same conditions and, unlike some of the simplified analogues, did not present tolerability issues in mice, although it lacked activity against *H. contortus*. These compounds could be regarded as simple amphiphiles, which are able to disrupt biological membranes and protein–protein interactions by virtue of their detergent-like physicochemical properties. However, the reported jietacins displayed some striking *regio*-isomerism and chiral effects, which would be difficult to explain if they acted as simple detergents. The vinyl azoxy group, in particular, appears to be worth investigating in other structural contexts.

## Recent patent literature on nematocides

**Fig. 10:**
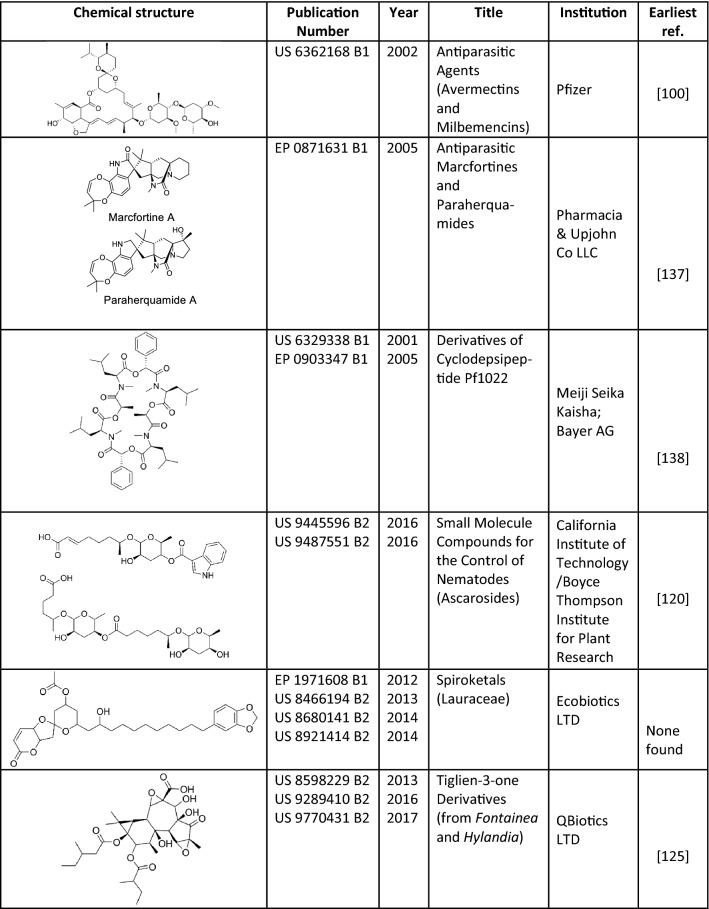
Patented nematocidal natural products (2000–2018)

There is a widespread belief that publications arising from industrial laboratories disclose projects that are no longer of interest to these organisations, casting doubt on the developability of published molecules, or at least on their commercial viability. This may not necessarily be the case, as at times pharmaceutical management considers it advantageous to increase their scientific profile through publications in open forums. In these situations, the chemical class of interest and its potential uses are patented before publication. Therefore, it could be expected that compounds with real drug development potential would appear in the patent literature before being reported in scientific journals. Academic research groups file patents much less frequently due, in large part, to the strong pressure to publish quickly and abundantly. This makes it difficult to gauge the real applied interest of research findings presented in peer-reviewed journals only, as they can be published either because they are not considered actionable, or because authors and their institutions are not willing to invest in IP protection. We searched the patent literature through SciFinder and “The Lens”, a comprehensive public database developed by Cambia and Queensland University of Technology [119], for patents granted on anthelmintics or nematocides since 2000, and manually filtered the results for natural products with disclosed activity against parasitic nematodes of animals. The end product was a list of 40 patents, which collectively covered the seven structural classes exemplified in Fig. 10. It is apparent that none of the structures reviewed here from the scientific literature, dating from 2010, has yet received patent protection. This would indicate a lack of commercial interest, but some compounds in Fig. 10 stem from natural product classes whose founding member was published decades before a patent was filed on an analogue structure. These patents disclose analogues or non-obvious derivatives of previously published structures, illustrating the possibility of using existing information to jump-start discovery projects that lead to novel IP.

Of note, all natural nematocides patented since 2000 and already in the market are derivatives of microbial products. They include the macrocyclic lactones, avermectins and milbemycins (e.g. generic abamectin, ivermectin and moxidectin,and proprietary brands such as Ivomec, Mectizan or Acarexx). There are also commercially available semi-synthetic versions of the *spiro*-oxindole paraherquamide (derquantel) and of the PF1022 cyclodepsipeptide (emodepside). Compounds disclosed in the most recent granted patents do not yet have commercially developed versions. The ascarosides are scientifically interesting starting points, as they could become the first commercial anthelmintics based on the structure of a nematode pheromone [120], although it is unclear, at this time, whether that particular mode of action will provide a potent enough deworming effect. Additional development issues of ascarosides may be the metabolic liabilities of their numerous hydroxyl groups and the fact that each nematode species seems to recognise a unique blend of different compounds [121, 122], raising the question as to how complex a mixture may need to be developed to reach an acceptabl activity spectrum.

Spiroketals are described in the listed patents as products extracted from plants of the family Lauraceae (*Litsea leefeana*, *Cinnamomum laubatii* and *Cryptocarya lividula*) from the Australian tropical forest. No public record was found in our searches indicating that they are being actively pursued as anthelmintics, and the original IP owner (Ecobiotics Ltd) was merged back into the Qbiotics group in 2017. This latter company, although listed as the inventor of the newest anthelmintic family granted patent protection (the tigliane phorbol esters) (Fig. 10), seems focused on developing this series for the potentially more lucrative anti-cancer indication [123, 124]. These compounds would be an example of an “old” structure (originally described from traditional medicinal plants from Africa; [125]) that was rediscovered later in a different source (Australian species of *Hylandia* and *Fontainea*) and can still generate actionable IP. The development of modern synthetic methods for the chemical class (reviewed in [126]), and a thorough characterisation of their activity, generated enough novelty to enable patenting. Going forward, the metabolic stability of the ester groups in the structure will need to be investigated, as they could either be a metabolic liability or pro-drug elements that contribute bioavailability to the active moiety.

There are no signs in the published record that any natural nematocides protected since 2000, but not yet in the market, are being actively developed into commercially available anthelmintics. If they are not, reasons are likely to include the scientific and technical issues presented earlier in this section, as well as return-on-investment considerations. For low price areas, such as neglected diseases or agricultural use, the development path needs to be inexpensive and thus short, implying that the original hit compound must have many of the attributes of a drug from the start.

## Conclusions

Since the last, comprehensive review of natural products with activity against parasitic nematodes [23], there have been partial revisions on the parasiticidal activities of plant extracts, essential oils and some individual compounds therein [127–129], sometimes focusing on specific parasites [130] or on a particular compound class [131]. Here, we critically appraised the scientific and patent literature for all pure natural compounds active against any parasitic nematode of animals, or against the model nematode *C. elegans*, from 2010 to 2018, to gain a perspective on the abundance of natural chemical starting points available for anthelmintic drug discovery. The findings are somewhat disappointing, considering that natural products are a main source of novel drug scaffolds [29], especially for anti-parasitics and antimicrobials. Only one new chemical class has emerged from the scientific literature in the review period, namely that of the open nemadectin analogues [103], and very limited effort seems to have been devoted to exploring the SARs of previously described compounds, with the exception of the jietacins [117]. Most publications analysed are rather preliminary reports of compounds with low to moderate activity from sources not explored before for anthelmintic activity, but yielding in the end already known chemical structures. Compounding the problem, most reviewed compound structures fall within classes with promiscuous activity and dubbed “Pan Assay INterference compoundS” (PAINS) or “Invalid Metabolic Panaceas” (IMPs) [42, 132], because they keep being rediscovered as hit compounds in unrelated biological assays. This may be due to easy chemical reactivity, optical properties or to a capacity for non-specifically binding and aggregating macromolecules, especially when compounds are assayed at hundreds of micromolar or millimolar levels. Such unwanted properties should be investigated as part of the hit characterisation process for compounds with structural alerts [133], something that, admittedly, may be difficult to do without a biologically relevant target-based assay. An intriguing conclusion from this review has been that plants are the sources for most compounds discussed herein. The only reported compound of fungal origin was β-sitosterol, also present in plants, while, arguably, the most interesting compounds, the open nemadectins (Macrocycles subsection) and the jietacins (Miscellaneous compounds subsection), were both isolated from cultures of *Streptomyces* species (bacteria). The overwhelming focus on plants seems to relate to ready accessibility, ethnobotanical considerations and the possibility of directly using plant material to treat helminthiases of animals. However, as discussed in the Background section, plants often present issues of irregular yields of active compounds and of sustainable supplies. Additionally, re-discovery rates seem to indicate that most major classes of plant products have already been described. Minor components of plant extracts with potent biological activity are probably still waiting to be isolated, but we appear to have reached a situation in which diminishing returns demand increased analytical efforts. The apparent rareness of screening activity using compounds from animal sources can be justified by the difficulties and costs associated with accessing their sources, but it is more difficult to explain why microbial extracts are not more widely reported in anthelmintic screens. Of the four novel anthelmintic classes introduced into general use since 2000, three originated from bacterial products (macrocyclic lactones, cyclooctadepsipeptides and paraherquamides), and only one is a fully synthetic class, the aminonitriles (monepantel). From a theoretical perspective as well, the genetic diversity, and, consequently, the metabolic capabilities of microbes far exceed those of metazoans [134], and there is a plethora of well-curated collections of pre-fractionated microbial extracts and pure compounds available for screening (cf. [37]), with far fewer reproducibility and sustainability issues than plant extracts. Therefore, the published literature leads to the conclusion that most research activity in this area is biased towards plant compounds primarily for reasons other than past or theoretical success rates. It may be that extracting plant material requires less infrastructure and know-how than isolating and culturing microorganism, and there is also the issue of research funding. Accessing well-curated natural products libraries involves some costs and compound screening is often derided as a “fishing expedition” and usually dismissed by research funding agencies, while the same work is considered too preliminary and risky for industry funding, thus limiting anthelmintic research to low-cost activities. It would help academic initiatives in this area if freely accessible high-throughput phenotypic assays were made widely available, as it has been done for antimicrobials [135]. Hopefully, the recognition of key helminthiases as neglected tropical diseases [136] should provide a much-needed boost to the field, before drug resistance renders current control activities ineffective for human patients as well as for livestock animals.

## Data Availability

All data discussed have been previously published.
